# Partial Right Atrial Inflow Occlusion for Transient Systemic Hypotension During Deployment of Thoracic Stentgrafts

**DOI:** 10.1007/s00270-020-02758-1

**Published:** 2021-03-11

**Authors:** L. C. Pietersen, R. W. van der Meer, D. J. C. Alders, J. van Schaik, D. Eefting, C. S. P. van Rijswijk

**Affiliations:** 1 Department of Interventional Radiology, Leiden University Medical Center, Albinusdreef 2, 2333 ZA, Leiden, The Netherlands; 2 Department of Anesthesiology, Leiden University Medical Center, Leiden, The Netherlands; 3 Department of Vascular Surgery, Leiden University Medical Center, Leiden, The Netherlands

**Keywords:** Right atrial inflow occlusion, Thoracic endovascular aorta repair, Occlusion balloon

## Abstract

**Purpose:**

Temporary balloon occlusion of the inferior vena cava to lower cardiac output is a relatively infrequently used technique to induce controlled systemic hypotension. In this technical note, we describe the feasibility, reliability, and safety of partial occlusion of right atrial inflow and the effect on systemic blood pressure during the deployment of a thoracic stentgraft.

**Materials and Methods:**

Twenty consecutive patients undergoing thoracic endovascular aortic repair, with proximal landing in zone 0–3 of the thoracic aorta, were prospectively included. Right atrial inflow occlusion was performed with a compliant occlusion balloon.

**Results:**

Median time to reach a mean arterial pressure of 50 mmHg was 43 s. Median recovery time of blood pressure was 42 s.

**Conclusion:**

Partial right atrial inflow occlusion with an occlusion balloon is feasible with reliable results and without procedure-related complications.

## Introduction

Precise positioning of a thoracic stentgraft is essential for technical and clinical success in endovascular aortic repair (TEVAR). Malpositioned or inaccurate stentgraft deployment may be caused by the pulsatile aortic blood flow potentially resulting in displacement of the stentgraft distally. Concurrently, the more TEVAR indications are extended to hemodynamic and anatomically challenging landing zones, and the more inaccurate stentgraft deployment becomes an issue. For this reason, several techniques to manage this downstream migration have been described including pharmacologically induced systemic hypotension, rapid right ventricular pacing, and recently the Munich Valsalva Implantation Technique (MuVIT) [1–3]. The technique of partial atrial inflow occlusion to rapidly achieve reversible systemic hypotension has been described previously by few other authors in animal studies and case series [4–6] and in one patient cohort study [7], but detailed information about the blood pressure over time during this maneuver is lacking. Temporary balloon occlusion of the inferior vena cava to lower cardiac output is a relatively infrequently used technique to induce systemic hypotension and may improve adequate stent placement [5, 6]. In this technical note, we describe our clinical experience with partial occlusion of right atrial inflow and the effect on systemic blood pressure during the deployment of a thoracic stentgraft in a large patient group.

## Material and Methods

Between January 2017 and March 2020, all consecutive patients undergoing TEVAR with proximal landing in zone 0–3 of the aorta according to Ishimaru’s classification scheme [8] were prospectively included. Individual informed consent or formal evaluation by an ethics committee is not required according to the Dutch law for scientific research on data gathered for the purpose of clinical patient care. All procedures were performed under general anesthesia. Heparin was administered during the procedure. Hemodynamic parameters were continuously measured using a peripheral arterial line, and a 3-lead ECG was recorded. Exact timing (in seconds) from the moment of balloon inflation, reaching mean arterial pressure of 50 mmHg, to the moment of balloon deflation, and recovery time of blood pressure to baseline were noted. Complications related to the atrial balloon inflation were registered.

Ultrasound-guided percutaneous arterial access or open exposure for the thoracic stentgraft was used, dependent on the indication for TEVAR and experience of the operator with the use of large bore vascular closure devices. For balloon occlusion of the vena cava inferior, either the ipsilateral (*n *= 7) or contralateral (*n* = 13) common femoral vein, compared to stentgraft introduction site, was chosen to introduce a 12 or 14 French introducer sheath for the balloon catheter. An open ipsilateral (*n* = 3) introduction of the balloon catheter was only considered if an open introduction of the stentgraft was chosen. In most of the patients (*n* = 17), a percutaneous introduction of the balloon catheter was chosen as manual compression of the venous puncture site is easy to perform.

**Fig. 1:**
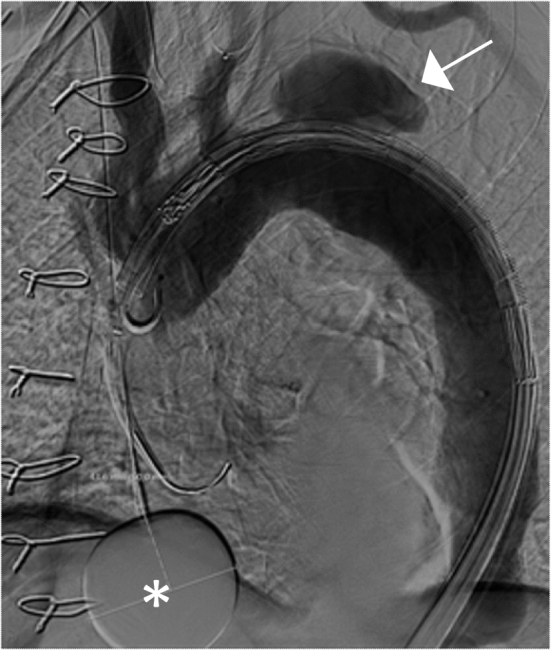
Angiogram showing an inflated occlusion balloon (asterisk) for partial occlusion of right atrial inflow in a patient with a false lumen aneurysm (arrowhead). The intended proximal landing is zone 2 covering the left subclavian artery

**Fig. 2:**
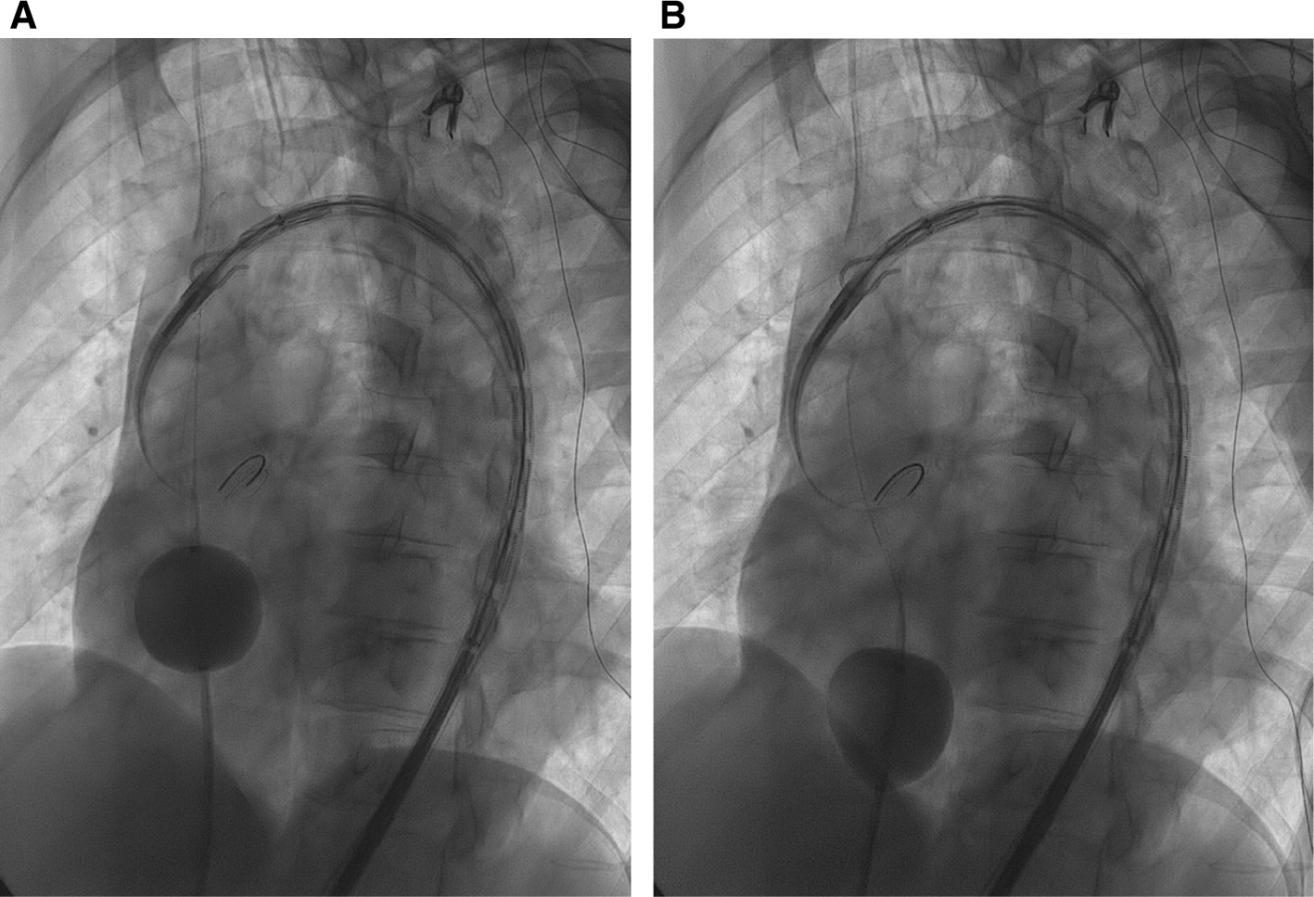
Fluoroscopic image (**A**) demonstrates an inflated compliant occlusion balloon in the right atrium with a mixture of iodine contrast and saline. Fluoroscopic image (**B**) demonstrates the retracted and deformed balloon at the atriocaval junction

A compliant occlusion balloon (Reliant Medtronic, Santa Rosa, CA, USA or CODA COOK Incorporated, Bloomington, IN, USA) was advanced into the right atrium and manually inflated with approximately 30 ml of iodine contrast and saline mixture (1:3) after stentgraft insertion into the desired position and angiography has been performed (Fig. 1). The balloon was retracted into the atriocaval junction to prevent inflow from the inferior vena cava into the right atrium under fluoroscopy. Correct positioning was confirmed by deformation of the inflated balloon (Fig. 2). The inflated balloon was kept in position under mild traction. When mean arterial pressure reached 50 mmHg, the thoracic stentgraft was completely deployed followed by immediate deflation of the occlusion balloon. After completion of the procedure, the venous femoral introducer sheath was removed and hemostasis was achieved by either surgical closure or by manual compression for 10 min when a percutaneous access was used.

Stentgraft migration was evaluated on post-procedural CT examinations performed within 30 days after TEVAR.

## Results

In twenty patients, mean age 70 years (range 43–83 years), 12 male and 8 female, TEVAR was performed using temporary balloon occlusion of the inferior vena cava. Most relevant comorbidities are presented in Table 1. In 1 patient (5%) the stentgraft was deployed in zone 0, in 1 patient (5%) in zone 1, in 5 patients (25%) in zone 2 and in 13 patients (65%) in zone 3. The indication for TEVAR was atherosclerotic aneurysmal disease (*n* = 8), acute complicated aortic dissection due to rupture (*n *= 2) or visceral ischemia (*n* = 1), chronic complicated aortic dissection with false lumen dilatation (*n* = 8) and peri-prosthetic bleeding after surgical arch repair (*n* = 1) (Table 1).

**Table 1:** Patient characteristics

	*n* = 20
Gender	Male *n* = 12: Female *n* = 8
Hypertension	*n* = 17
Heart disease	Non ischemic cardiomyopathy (*n* = 2), ischemic coronary disease (*n* = 4)
Proximal sealing zone	0 (*n* = 1) 1 (*n* = 1) 2 (*n* = 5) 3 (*n* = 13)
Indication	Chronic dissection with false lumen aneurysm (*n* = 8) Acute dissection with visceral ischemia (*n* = 1) Peri-prosthetic bleeding after surgical arch repair (*n* = 1) Acute complicated aorta dissection with rupture (*n* = 2) Atherosclerotic aneurysm (*n* = 8)
Thoracic stentgraft	Valiant Captivia (*n* = 3), Medtronic* Custom-made fenestrated arch graft (*n* = 2), Zenith TX2 (*n* = 10), Zenith Alpha (*n* = 5), COOK**
Length of proximal stentgraft	Median 202 mm (range 150–217 mm)
Occlusion balloon	Reliant balloon catheter, Medtronic* compatible with 12 French introducer sheath (*n* = 14) CODA balloon catheter, COOK**compatible with 14 French introducer sheath (*n* = 6)
Venous access	Open (*n* = 3) Percutaneous (*n* = 17)

*Medtronic, Santa Rosa, CA, USA

**COOK Incorporated, Bloomington, IN, USA

Median time to reach a mean arterial pressure of 50 mmHg was 43 s (range 8–137 s). Median recovery time of blood pressure was 42 s (range 12–320 s). The median inflation time of the balloon was 113 s (range 54–196 s). One patient did not show decrease in blood pressure after repeated inflation and positioning of the occlusion balloon. Three patients demonstrated ECG abnormalities (arrhythmia and/or ST depression) during inflation of the occlusion balloon which recovered spontaneously after deflation of the balloon. The ECG abnormalities occurred in two patients with proximal stentgraft landing in zone 3 and in one patient in zone 2. ECG abnormalities may have been related to wire positioning against the aortic valve. No venous bleeding or thrombotic complications occurred, and no arterial stentgraft access problems were noted. In 19 patients, accurate positioning of the stentgraft on the intended proximal landing site could be achieved. None of these patients showed a type 1A endoleak.

Discrepancy between the planned and achieved landing sites during stentgraft deployment occurred in 1 of 20 patients (zone 3). Stentgraft migration was 12 mm and could be explained by an extreme steep arch. Despite the choice for the Zenith TX2 COOK stentgraft with Pro-Form (which is designed with controlled shortening and overlap of the first two proximal stents that should result in greater conformability and improved wall apposition), migration occurred due to the inability of the stentgraft to conform to the inner curvature of the aortic arch. In this patient, a second stentgraft was placed to extent the covered part of the aorta proximally. After placement of the second stentgraft, no type 1A endoleak was visualized.

## Discussion

In this patient cohort study, we described our clinical experience with temporary balloon occlusion of the right atrium to decrease cardiac output during TEVAR. We showed that temporary balloon occlusion of the right atrium is a reliable, safe and simple technique. Depending on the proximity of the planned thoracic stentgraft, controlled hypotension with reduction of the cardiac output to reduce the impact of blood flow on positioning may be helpful to limit stent migration. We found a median time to reach a mean arterial pressure of 50 mmHg of 43 s with recovery to the initial blood pressure in 42 s, a bit longer compared to the findings of Marty et al. who reported the time of the complete partial inflow occlusion maneuver of less than one minute [7]. This technique induces systemic hypotension through a significant reduction of cardiac preload and can be rapidly reversed by the operator. Rapid recovery of systemic blood pressure is needed to reduce the risk of complications such as ischemic stroke and spinal cord ischemia. This technique can relatively safely be applied in patients with ischemic heart disease because it does not increase myocardial oxygen demand during induced hypotension, in contrast to increased oxygen consumptions when using rapid pacing [9, 10].

Rapid ventricular pacing using transvenous pacing wires is another invasive method for inducing systemic hypotension and reducing left ventricular output mainly performed during transcatheter aortic valve implantation (TAVI). This technique is reliable and rapid but needs a cardiologist or cardio-anesthesiologist. Studies have shown the advantage of rapid pacing over pharmacologically induced hypotension by significantly faster decrease in blood pressure to target level with significantly shorter restoration time [11], but procedure-related complications and deaths have been reported [12].

Modulation of systemic blood pressure is typically achieved using intravenous medication. Most agents used have a relatively rapid onset of action and a short half-life of several minutes. However, in practice, an individualized dose is needed in different patients. Therefore, it is less controllable compared to induced hypotension by partial right atrial inflow. Moreover, pharmacologically induced hypotension, e.g., with Nitroglycerin, may result in compensatory tachycardia.

Recently, the MuVIT technique has been published by Tsilimparis et al. [3] describing reduced cardiac output by a modified Valsalva maneuver due to controlled manual ventilation performed by the anesthesiologist. This technique needs to be evaluated but seems promising and eliminates the need for additional venous access.

The use of a vena cava inferior occlusive balloon is an invasive procedure which requires additional venous access. However, we did not experience post-procedural bleeding or thrombotic complications. In one patient, a decrease in blood pressure was not accomplished after the deployment of the vena cava occlusive balloon. This concerned a 43-year-old woman who presented with a chronic type B aortic dissection. She had no known medical history and only used oral contraceptive medication. She received a low dose of norepinephrine after an aortic dissection was diagnosed. We do not have a clear explanation for this specific happening. Possibly the deployment of the occlusion balloon did not completely occlude the inferior vena cava. There were no peri-procedural complications. After adding nitroglycerine, a slight decrease in blood pressure was seen.

The contraindications for the use of an occlusion balloon are severe ischemic heart disease with unstable coronary artery disease or recent myocardial infarction. Relative contraindications are excessive risk of bleeding or severe hypotension.

Indications for endovascular treatment of the aortic arch and proximal descending thoracic aorta with stentgrafts are expending, and stentgraft design is improving. Despite this, technical and anatomic difficulties persist caused by the inflexible structure of most stentgrafts and their inability to conform to the curvature of the aortic arch, as seen in one of our patients.

In conclusion, right atrial inflow reduction is a relatively simple and safe interventional technique which can be used for quick and controlled reduction of the systemic blood pressure by lowering the cardiac output. This technique may be helpful for more exact positioning of a proximal thoracic stentgraft.
